# Staphylococcal TSST-1 Association with Eczema Herpeticum in Humans

**DOI:** 10.1128/mSphere.00608-21

**Published:** 2021-07-28

**Authors:** Patrick M. Schlievert, Richard J. Roller, Samuel H. Kilgore, Miguel Villarreal, Aloysius J. Klingelhutz, Donald Y. M. Leung

**Affiliations:** a Department of Microbiology and Immunology, Carver College of Medicine, University of Iowa, Iowa City, Iowa, USA; b Rho Federal Systems Division, Inc., Durham, North Carolina, USA; c Division of Allergy/Immunology, Department of Pediatrics, National Jewish Health, Denver, Colorado, USA; University of Kentucky

**Keywords:** CD40, *Staphylococcus aureus*, atopic dermatitis, chemokines, eczema, eczema herpeticum, epithelial cells, herpes simplex virus, staphylococcal enterotoxins, toxic shock syndrome toxin 1

## Abstract

Atopic dermatitis (AD) is a condition affecting 30 million persons in the United States. AD patients are heavily infected with Staphylococcus aureus on the skin. A particularly severe form of AD is eczema herpeticum (ADEH), where the patients’ AD is complicated by S. aureus and herpes simplex virus (HSV) infection. This study examined the S. aureus strains from 15 ADEH patients, provided blinded, and showed a high association of ADEH with strains that produce toxic shock syndrome toxin-1 (TSST-1; 73%) compared to 10% production by typical AD isolates from patients without EH and those from another unrelated condition, cystic fibrosis. The ADEH isolates produced the superantigens associated with TSS (TSST-1 and staphylococcal enterotoxins A, B, and C). This association may in part explain the potential severity of ADEH. We also examined the effect of TSST-1 and HSV-1 on human epithelial cells and keratinocytes. TSST-1 used CD40 as its receptor on epithelial cells, and HSV-1 either directly or indirectly interacted with CD40. The consequence of these interactions was chemokine production, which is capable of causing harmful inflammation, with epidermal/keratinocyte barrier disruption. Human epithelial cells treated first with TSST-1 and then HSV-1 resulted in enhanced chemokine production. Finally, we showed that TSST-1 modestly increased HSV-1 replication but did not increase viral plaque size. Our data suggest that ADEH is associated with production of the major TSS-associated superantigens, together with HSV reactivation. The superantigens plus HSV may damage the skin barrier by causing harmful inflammation, thereby leading to increased symptoms.

**IMPORTANCE** Atopic dermatitis (eczema, AD) with concurrent herpes simplex virus infection (eczema herpeticum, ADEH) is a severe form of AD. We show that ADEH patients are colonized with Staphylococcus aureus that primarily produces the superantigen toxic shock syndrome toxin-1 (TSST-1); however, significantly but to a lesser extent the superantigens staphylococcal enterotoxins A, B, and C are also represented in ADEH. Our studies showed that TSST-1 uses the immune costimulatory molecule CD40 as its epithelial cell receptor. Herpes simplex virus (HSV) also interacted directly or indirectly with CD40 on epithelial cells. Treatment of epithelial cells with TSST-1 and then HSV-1 resulted in enhanced chemokine production. We propose that this combination of exposures (TSST-1 and then HSV) leads to opening of epithelial and skin barriers to facilitate potentially serious ADEH.

## INTRODUCTION

Atopic dermatitis (AD) is a chronic skin condition affecting millions of children worldwide, extending into adulthood (1). A complication of AD is the presence of concurrent herpes simplex virus (HSV) infection, a condition termed eczema (atopic dermatitis) herpeticum (ADEH). ADEH can be severe (2, 3).

Previously, we studied the association of Staphylococcus aureus and its superantigens (SAgs) with AD (4, 5) and other unrelated conditions, for example, cystic fibrosis (6) and various forms of toxic shock syndrome (TSS) (7–9). It has been well established that 100% of pathogenic S. aureus isolates produce SAgs (1, 10). However, not all SAg serotypes are associated with these various conditions. For example, 100% of menstrual TSS cases are caused by S. aureus strains that produce the SAg TSS toxin-1 (TSST-1) (1, 7, 10, 11); they secrete few other protein virulence factors (12). In contrast, only approximately 10% of AD and cystic fibrosis isolates produce TSST-1 (6). There is no thorough explanation for the association of TSST-1 with menstrual TSS, except that this SAg has 10-fold-greater interaction with the immune coreceptor CD40 on vaginal epithelial cells than the other two major causes of TSS, notably staphylococcal enterotoxin serotypes B and C (SEs B and C) (13). This enhanced interaction with epithelial cells by TSST-1 through CD40 has been proposed to cause menstrual TSS through harmful inflammation with barrier disruption and downstream TSS (14). TSST-1, SEA, SEB, and SEC account for nearly 100% of nonmenstrual TSS (1, 10, 15). In the first description of postinfluenza TSS, 100% of the fatal cases were associated with TSST-1 S. aureus (9). This observation was also noticed by one of the authors (P. M. Schlievert) in multiple unpublished, blinded studies. The conclusion from the above studies is that TSST-1 may be more potent in causing TSS than SEB and SEC due to its stronger interaction with CD40 on both human epithelial cells and keratinocytes (13, 16). The downstream effect of TSST-1 may be to induce significant chemokine production by epithelial cells and keratinocytes (for example, interleukin-8 [IL-8] and MIP-3α), leading to harmful inflammation and opening of the barriers to additional SAg penetration (14, 17). However, TSST-1, SEB, and SEC also appear to be significant causes of TSS, including fatal cases, possibly because of the exceptionally high level of their production in biofilms (18); only occasionally can SEA be produced in high-enough concentrations to cause TSS (4, 15).

For typical AD isolates from patients without EH and those from the unrelated condition cystic fibrosis, there appears to be overrepresentation of the enterotoxin gene cluster compared to isolates from other sources (5, 6). In both AD and cystic fibrosis, only approximately 10% of S. aureus isolates produce TSST-1; these same isolates produce the enterotoxin gene cluster of six SAgs, and an additional large number of isolates produce the enterotoxin gene cluster without TSST-1 (5, 6). The enterotoxin gene cluster of SAgs, produced only in small amounts, appears to be important for human colonization as opposed to production of TSS (19).

In menstrual TSS, it was first proposed that HSV might be the cause (20). It was later shown that TSST-1 is the cause (7), but presumably there is some type of immune dysregulation which allows HSV to reactivate in menstrual TSS patients. This might result from several factors, including association of menstruation itself with HSV reactivation and induction of a cytokine storm by TSST-1 (14) that might compromise immune control of HSV reactivation or possibly induce virus reactivation in latently infected neurons directly or indirectly by cytokine-induced fever (21).

This study was undertaken to assess SAg production by ADEH S. aureus compared to recently published data on SAg production by S. aureus infections in other human conditions. We show that the majority of ADEH S. aureus isolates produce TSST-1, with nearly all of the remaining isolates producing SEB or SEC; two additional isolates produced SEA in higher-than-usual concentrations. TSST-1 production, and indeed production of all of these TSS-associated SAgs (TSST-1, SEA, SEB, and SEC), is overrepresented in ADEH compared to other S. aureus isolates from other conditions. We provide possible explanations for the high association.

## RESULTS

A total of 15 ADEH S. aureus isolates were available for testing from the NIAID Atopic Dermatitis Research Network (ADRN). These isolates, from lesional skin of independent patients, were evaluated in blinded fashion for the presence of the genes and for production of TSST-1, SEB, and SEC, the major SAgs associated with TSS. For TSST-1, 11/15 (73%) isolates had the ability to produce this SAg (Table 1). The average amount of TSST-1 produced in broth cultures ± standard deviation (SD) was 5.7 ± 1.9 μg/ml. Two of these 11 isolates concurrently had the ability also to produce SEC, and one of the 11 isolates concurrently had the ability to produce SEB. If the percentage of TSST-1 S. aureus from ADEH patients is compared to recent isolates from two other conditions, typical AD (tested for both genes and proteins) and the unrelated condition cystic fibrosis (tested for genes), the numbers positive differed significantly with *P* < 1.2 × 10^−6^ and 2.8 × 10^−6^, respectively, as determined by Fisher’s exact test. Five ADEH isolates produced SEC (3 isolates) or SEB (2 isolates) (Table 1). If combined, the production of SEB and SEC, which are highly related SAgs (1, 10, 22), was 30.7 ± 9.4 μg/ml. Thus, 13/15 isolates produced one or more of the SAgs most highly associated with TSS (1, 10). There was a 1:1 correlation between the presence of the SAg gene and production of these SAg proteins in our study. The remaining two isolates, which were negative for production of TSST-1, SEB, and SEC, were positive for production of SEA, a SAg only occasionally associated with nonmenstrual TSS (15). Typically, SEA is produced in very small amounts, 75 pg/ml to 1 ng/ml as tested *in vitro* (4). The two isolates from ADEH, which were SEA positive in the absence of TSST-1, SEB, or SEC, produced SEA *in vitro* in broth cultures in amounts of 4.6 μg/ml, or approximately 4,500 times more than usually observed.

**TABLE 1:** SAg production by ADEH isolates of S. aureus compared to typical AD and the unrelated condition cystic fibrosis isolates

Superantigen(s)	Condition type (no. of isolates/total)	% positive
TSST-1	ADEH (11/15)	73^*a*^
	Typical AD (8/78)	10
	Cystic fibrosis (9/77)	12

SEB/SEC	ADEH (5/15)	33
	Typical AD (13/78)	17
	Cystic fibrosis (10/77)	13

a Significantly different from typical AD and cystic fibrosis S. aureus isolates with *P* < 1.2 × 10^−6^ (compared to typical AD) and 2.8 × 10^−6^ (compared to cystic fibrosis) with use of Fisher’s exact test.

The data indicate that ADEH isolates of S. aureus are highly and significantly overrepresented with ability to produce TSST-1 and to a significant but lesser extent to produce one of the other major SAgs associated with TSS (SEA, SEB, and SEC). Our prior studies have shown that TSST-1 and SEs B and C can be produced in concentrations of ≥15,000 μg/ml as might be expected in biofilms in AD lesion skin (18). This observation by itself could explain the severity of ADEH. The eczema area and severity index (EASI) for the 15 patients with ADEH was determined to be an average of 17.5, placing these patients in the moderately severe AD category.

It has previously been noted that menstrual TSS has associated immune dysregulation that allows HSV reactivation, such that it was originally proposed that TSS may be caused by HSV (1, 10, 20, 23). While TSS is caused by staphylococcal SAgs, it is possible that the presence of SAgs leads to increased virus-induced harmful inflammation or replication in ADEH patient epithelial cells and keratinocytes, which in turn leads to increased HSV infection of the same cells.

We have previously shown that SAgs cause proinflammatory chemokine production (both IL-8 and MIP-3α) by both human vaginal epithelial cells (HVECs) and human keratinocytes (13, 16). We have also previously shown that TSST-1 (and SEs B and C) uses CD40 as its only receptor on HVECs. We have an isogenic pair of HVECs that differ only in CD40 surface expression; the CD40 was knocked out using CRISPR/Cas9 technology (13). We also have the complemented cell line with CD40 expressed on a plasmid.

**FIG 1:**
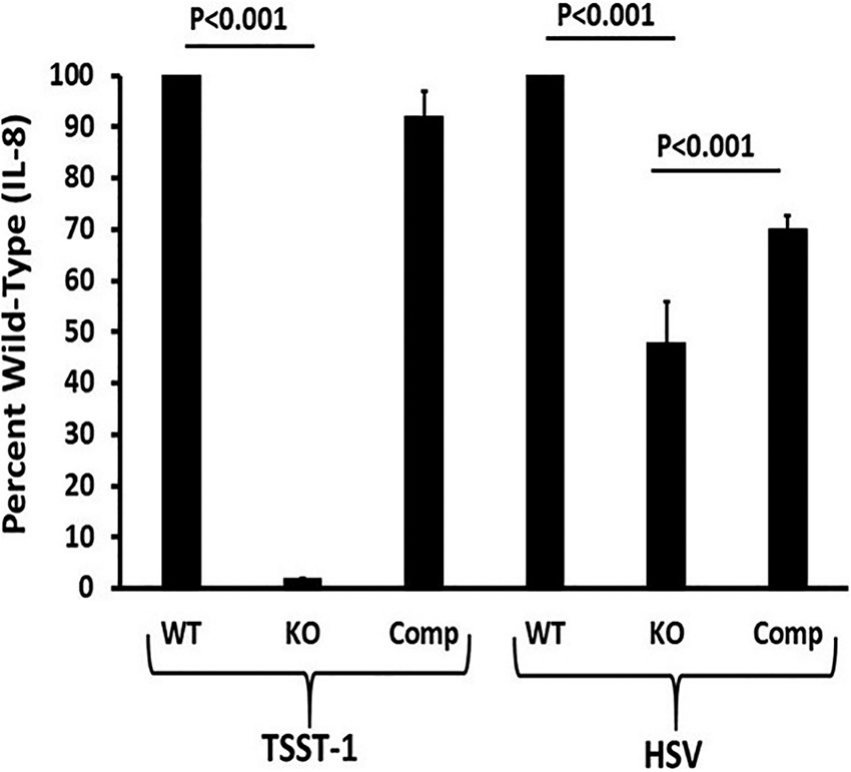
CD40 dependence of IL-8 production in TSST-1-treated and HSV-1-infected human vaginal epithelial cells (CD40 wild type [WT], CD40 CRISPR/Cas9 knockout [KO], and HVECs complemented with CD40 on a plasmid [Comp]). Human vaginal epithelial cells were cultured in triplicate in 96-well flat-bottom tissue culture dishes until confluent. Then, the medium was changed to keratinocyte serum-free medium (KSFM) with TSST-1 or HSV-1. Incubation was continued for 6 additional h, and the plate was frozen at −20°C to lyse cells. Subsequently, IL-8 concentrations in supernatants were determined by enzyme-linked immunosorbent assay (ELISA). Data are reported as means ± SD as a percentage of wild-type value.

Our studies confirmed that TSST-1 used only CD40 as its receptor to produce chemokines such as IL-8 (attractant of neutrophils), as tested in the present studies (Fig. 1). We have performed similar studies to assess the chemokine MIP-3α (signal for tissue damage and attractant of many immune cell types), with similar results (13). To determine whether CD40 mediates chemokine production by HSV-1-treated HVECs, we compared production levels of IL-8, as a representative chemokine, in wild-type, CD40 knockout, and CD40-complemented cells (Fig. 1). We do not have an isogenic pair of cell lines of keratinocytes, but our transcriptome sequencing (RNAseq) studies showed that TSST-1 and SEB caused the upregulation of nearly all components of the CD40 pathway leading to chemokine production (16). It is thus possible and appears likely that HVECs and human keratinocytes respond comparably. Our studies showed that IL-8 expression is diminished by about 50% in HSV-1-treated CD40 knockout cells compared to the wild type, and this effect is partially reversed by complementation. We used percent reduction compared to wild-type HVECs since the values of IL-8 produced vary from day to day. Typically, TSST-1 and HSV-1 alone induced 100 to 300 pg/ml of IL-8 from wild-type HVECs. The reduction of IL-8 production by the CD40 knockout cells was consistently near 50% regardless of experiment. In contrast, as shown previously, latex beads and normal vaginal flora Enterococcus faecalis did not induce IL-8 production from wild-type HVECs (24, 25). This suggests that CD40 is at least partially responsible for promoting chemokine expression in HSV-1-treated cells.

**FIG 2:**
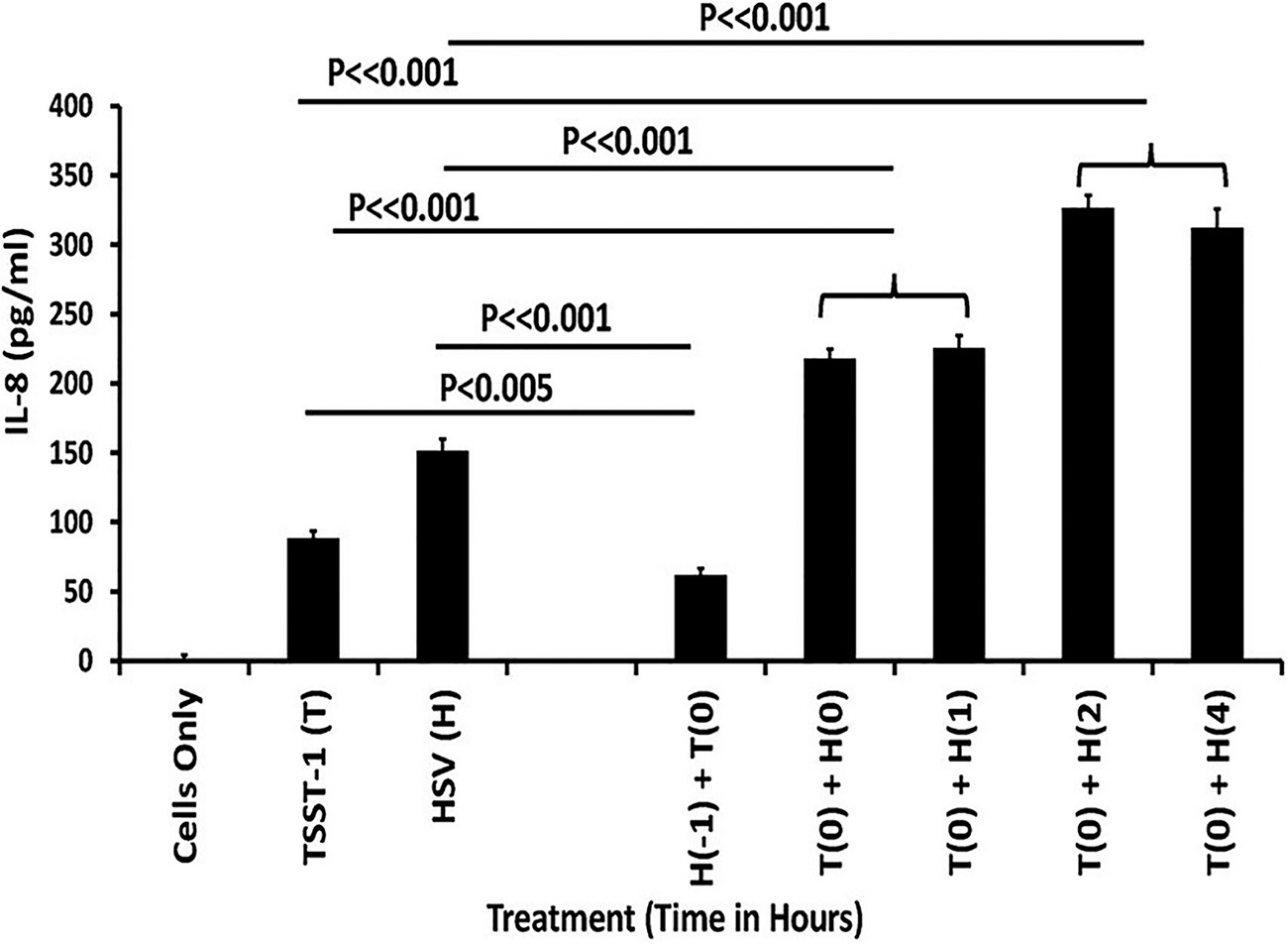
TSST-1 followed by HSV-1 induces chemokine production (IL-8) by human keratinocytes. Human keratinocytes were incubated until confluent in 96-well flat-bottom tissue culture plates in KSFM. Subsequently, the medium was changed and TSST-1 (T) and HSV-1 (H) were added to wells in triplicate as indicated. The numbers in parentheses indicate time in hours before or after treatment with TSST-1. The cells were incubated for a total of 6 h after time zero at 37°C, 5% CO_2_. Then, the plate was frozen at −20°C to lyse cells. IL-8, as a representative chemokine, was then measured by ELISA. Data reported as means ± SD.

The data suggested the possibility that HSV-1 synergizes with TSST-1 to cause human epithelial cells to produce chemokines. As shown in Fig. 2, when TSST-1 was administered prior to HSV-1, the combination of agents synergized to lead to IL-8 production. This production has been previously proposed to lead to harmful inflammation and opening of the skin (or mucosal) barrier to additional infection (14, 24, 26) by both bacteria (S. aureus) and viruses (HSV-1, simian immunodeficiency virus [SIV]). When HSV-1 was administered prior to TSST-1, the synergy was not observed (Fig. 2). These data make sense consistent with the appearance of HSV-1 in TSST-1-induced TSS. That is, TSST-1 causes immune dysregulation to facilitate reactivation of HSV-1, and the two molecules/particles synergize through harmful inflammation to disrupt the skin barrier, leading to additional AD symptoms and possibly more serious disease.

**FIG 3:**
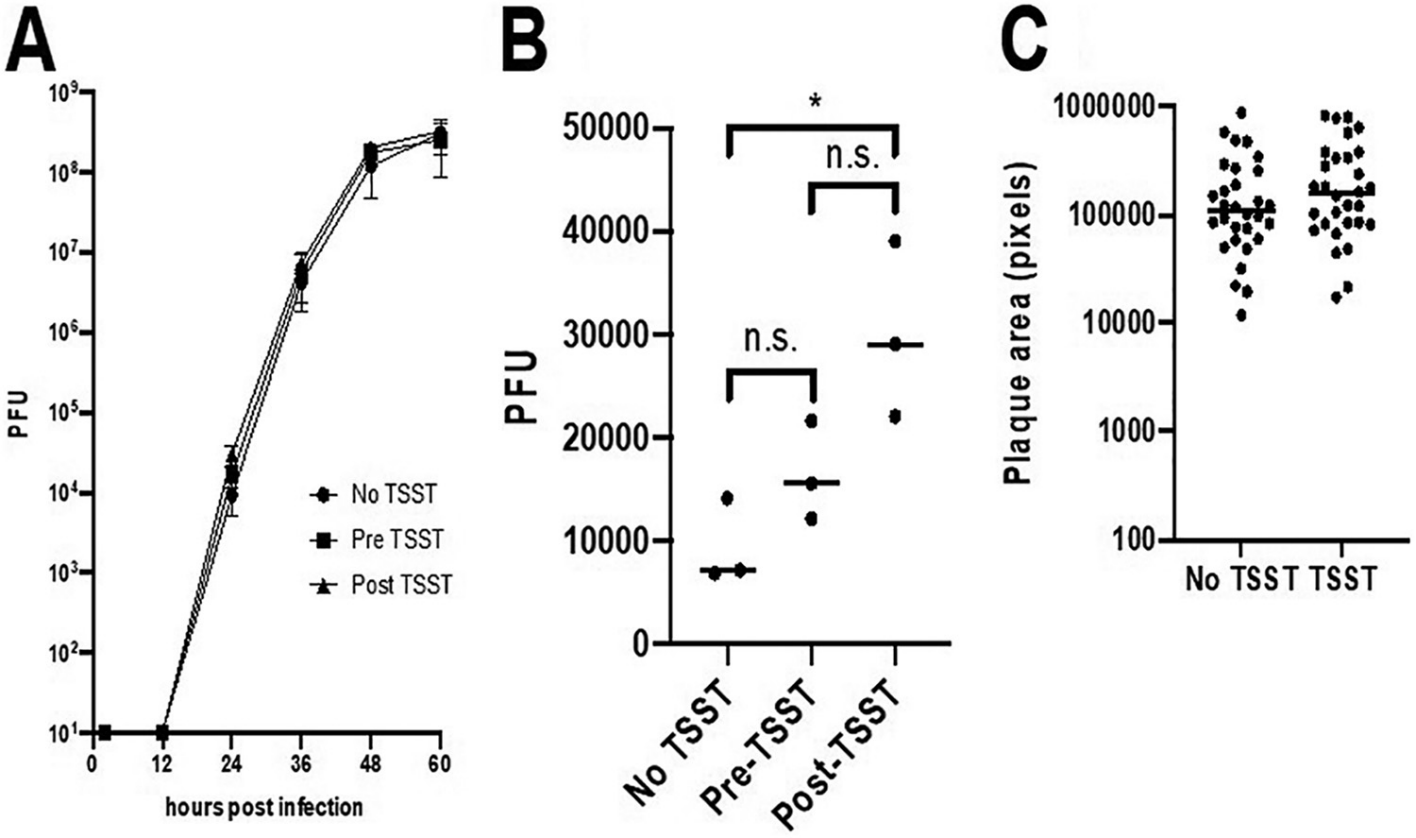
TSST-1 effects on HSV-1 growth and spread in keratinocytes. (A) Multistep growth of HSV-1(F) on HaCaT keratinocyte cultures that were untreated (No TSST) or treated with 10 μg/ml TSST-1 beginning 1 h before initiation of infection (Pre-TSST) or 2 h after initiation of infection (Post-TSST). (B) Data from the experiment shown in panel A at 24 h after initiation of infection demonstrated enhanced HSV-1 production after TSST treatment (*P* < 0.05). n.s., not significant. (C) Spread of HSV-1(F) on HaCaT cells treated with 10 μg/ml TSST-1 beginning 2 h after initiation of infection.

Finally, we examined whether or not prior TSST-1 administration would increase infection and/or spread of HSV in epithelial cells in multistep growth and plaque size assays performed on the HaCaT immortalized keratinocyte cell line (Fig. 3). Treatment of HaCaT cells with TSST-1 beginning 1 h before infection with HSV-1 (pre-TSST) had no significant effect on viral yield at any time after infection (Fig. 3A). TSST-1 treatment beginning 2 h after initiation of infection was associated with a small but statistically significant elevation of viral yield at 24 h (Fig. 3B), but this effect was not observed at later time points. Neither treatment affected plaque size (Fig. 3C). These data suggest that TSST-1 does not enhance viral replication or spread in immortalized keratinocytes.

## DISCUSSION

AD is a skin condition that affects more than 30 million Americans. All or nearly all of these patients have damaged or barrier-deficient skin (2, 27) that leads to concurrent S. aureus infections (1). The S. aureus strains that infect AD patients are quite diverse clonal groups, although strains producing the six-membered enterotoxin gene cluster of SAgs are highly overrepresented (4, 5). These enterotoxin gene cluster SAgs are considered important for colonization as opposed to TSS production (19). It has also been shown that staphylococcal alpha-toxin may enhance viral infection of cells, contributing to pathology (28). Many skin S. aureus isolates produce significant amounts of alpha-toxin (12).

The most important finding in this study is the high association of ADEH with the SAg TSST-1 and significantly but to a lesser extent the other SAgs, SEA, SEB, and SEC, all three associated with TSS (1, 10, 15). It is important that certain kinds of human mucous membrane and skin diseases are very highly associated with TSST-1, and secondarily SEB and SEC, two SAgs that are 75% identical and immunologically cross-reactive (22); only occasional TSS cases are associated with SEA (15). Menstrual TSS is 100% associated with TSST-1 (7, 11). Postinfluenza TSS is primarily associated with TSST-1 and somewhat with SEB/SEC (9). Nonmenstrual TSS is 50% associated with TSST-1 and nearly 50% with SEB/SEC (8). Cutaneous T cell lymphoma (CTCL) is associated with infection also approximately 50% by TSST-1 S. aureus (29); other SAgs are associated also with CTCL. Finally, 88% of bullous pemphigoid patients are lesion-infected with TSST-1 S. aureus (K. N. Messingham, M. Cahill, S. H. Kilgore, A. Munjal, P. M. Schlievert, and J. A. Fairley, submitted for publication). Collectively, these data suggest that certain niches in humans in association with certain conditions are highly associated with a unique SAg or unique SAg cluster.

ADEH is a relatively uncommon but potentially severe form of AD (2, 3). The current study surprisingly showed that the S. aureus isolates from ADEH patients are fairly clonal in nature, with strains producing primarily the SAg TSST-1, with smaller numbers producing SEB/SEC; two additional isolates produced SEA at higher-than-usual concentrations. S. aureus isolates that produce TSST-1 belong to clonal group USA200 (also known as CC30) (11). These strains have the additional unique property of producing little if any staphylococcal alpha-toxin (12, 30); the strains also typically produce the enterotoxin gene cluster of six colonization SAgs (11). The lack of or high reduction of alpha-toxin production by the TSST-1-producing isolates suggests ADEH does not depend on that toxin; the major human effect of alpha-toxin is causing dermonecrosis of skin. Additionally, a wide variety of S. aureus strains today produce the enterotoxin gene cluster, which includes SAgs that are thought to facilitate colonization and not enhancement of infection; these latter SAgs are produced only in minute quantities, insufficient to cause immune dysregulation systemically as seen in TSS (1, 19). USA400 (CC1) isolates continue to be common in many parts of the United States. These isolates nearly always produce SEB and/or SEC (31, 32). Like TSST-1, and as shown in our studies, these isolates produce SEB/SEC in high concentrations.

One limitation of the current study is that we had 15 ADEH isolates for testing, compared to 78 for typical AD and 77 for cystic fibrosis. All 15 of the ADEH isolates were from lesional skin. Although it would have been stronger to have more ADEH isolates, these were the strains available through the ADRN. However, statistically the presence of the TSST-1 gene and protein was very highly associated with ADEH, compared to typical AD and cystic fibrosis. The *P* values for significant differences were in the 10^−6^ range, making it highly unlikely that the data obtained were by chance. It also helps that the ADEH isolates were tested in blinded fashion to help avoid bias.

Having noted the high association of ADEH with TSST-1 and SEA/SEB/SEC, we thus set out to attempt to explain the fairly unique association of ADEH with TSST-1 and to a lesser extent SEA/SEB/SEC. It has previously been shown that patients with menstrual TSS, which is 100% caused by TSST-1 (11), also have concurrent HSV infections (20). Indeed, it was originally postulated that HSV may be the cause of menstrual TSS (20). It is now believed that the immune system dysregulation caused by TSST-1, as seen in menstrual TSS patients, is responsible for reactivation of latent HSV (11). It thus seems reasonable that ADEH occurs as a result of TSST-1, and to a lesser extent SEASEB/SEC, causing significant immune system dysregulation with consequent reactivation of latent HSV.

We previously showed that TSST-1 may be uniquely associated with menstrual TSS because of its high interaction with the immune costimulatory molecule CD40, the only receptor on human vaginal mucosal cells for SAgs (13). We have previously reviewed the putative mechanism downstream of CD40 interaction (14). The sequence is as follows: (i) TSST-1 interaction with CD40 leads to production of many proinflammatory chemokines (33), including IL-8 and MIP-3α; we have recently shown by RNAseq that TSST-1 and SEB likewise activate production of many proinflammatory chemokines in primary human keratinocytes (16); (ii) the proinflammatory chemokines attract polymorphonuclear cells and cells of the adaptive immune system which function to cause harmful inflammation to open the mucosal barrier further, allowing greater transport of TSST-1 across the barrier; and (iii) TSST-1 activates T cells and macrophages through cross-bridging of the variable region β-chain 2 of the T cell receptor with major histocompatibility complex class II (MHC II) on macrophages, with the downstream effect being a cytokine storm called TSS (1, 14, 34).

This study provided evidence that supports this sequence of events in ADEH. First, we showed that nearly all S. aureus isolates from ADEH patients produce TSST-1 or SEA/SEB/SEC individually or in combination. The higher association of ADEH with TSST-1 than with SEA/SEB/SEC may be the result of the greater effects of TSST-1 on CD40 to induce chemokine production, selection of isolates that produce this SAg, and consequent barrier disruption (13, 16). Second, we showed that both TSST-1 and HSV may use CD40, in total for TSST-1 and in part for HSV, to cause chemokine production. Third, the downstream effect of first exposure of epithelial cells to TSST-1 and then HSV is the augmented production of chemokines by these cells. Exposure of cells to HSV first followed by TSST-1 leads to suppression of chemokine production. Finally, in our recent RNAseq analysis of primary human keratinocytes, the HSV pathway of interaction with cells was significantly altered by TSST-1 and SEB (16). Although the exact significance of this finding is unclear, it suggests dysregulation. We hope subsequent studies will provide clarity.

It is important to consider that typically the first human cells to encounter S. aureus and its major SAgs (TSST-1, SEA/SEB/SEC) to establish infections are epithelial cells on mucous membranes and keratinocytes in skin. Both cell types are responsible for barrier formation. We have shown previously that the epithelial barrier is disrupted by TSST-1, and to a lesser extent SEB/SEC, in part through chemokine production by the epithelial cells and consequent induction of harmful inflammation (13, 14). These SAgs are produced in very high concentrations, achieving amounts as high as 15,000 μg/ml or more in biofilms (18), as opposed to other SAgs, which are produced in 10^4^ to 10^6^ μg/ml smaller amounts.

The interesting association of TSST-1- and SEA/SEB/SEC-positive S. aureus isolates with HSV infection in ADEH might, in principle, result from several effects other than chemokine augmentation and barrier disruption, including (i) enhanced replication or spread of HSV in infected epidermis due to activation of pathways that promote virus replication in susceptible cells, (ii) enhanced susceptibility to primary HSV infection due to compromised barrier function of the epidermis, (iii) enhanced symptoms of HSV infection due to recruitment of inflammatory cells to the site of infection, or (iv) enhanced reactivation of latent HSV infection in previously infected individuals induced by SAg expression. Our data address the first of these effects, showing that HSV infection and spread in cultured keratinocytes are somewhat enhanced by TSST-1 treatment. With regard to the second and third effects, TSST-1 and SEA/SEB/SEC can all cause induction of a local inflammatory response. As noted above, one consequence is compromised barrier function of the epidermis. Primary HSV infection of skin is strongly promoted by defects in the epidermal barrier, and therefore, infection by S. aureus that produces these toxins could strongly enhance susceptibility to HSV. Furthermore, the symptomatic manifestations of HSV infection of the skin can be considerably longer lasting and more severe in immunocompromised individuals, and the altered immune function induced by bacterial toxin expression might locally mimic the immunocompromised environment. Finally, HSV reactivation is known to be induced by several stimuli that could be created or enhanced by S. aureus skin infection and SAg action, including compromised immune surveillance at the site of latency, elevation of temperature, and even psychological stress. Clearly, TSST-1 and other major SAgs are considered potent pyrogens (1, 10), and TSS is defined by having high fever (35, 36). Furthermore, there are known nervous system dysfunctions in TSS (37), though the mechanisms underlying these dysfunctions remain to be determined.

**FIG 4:**
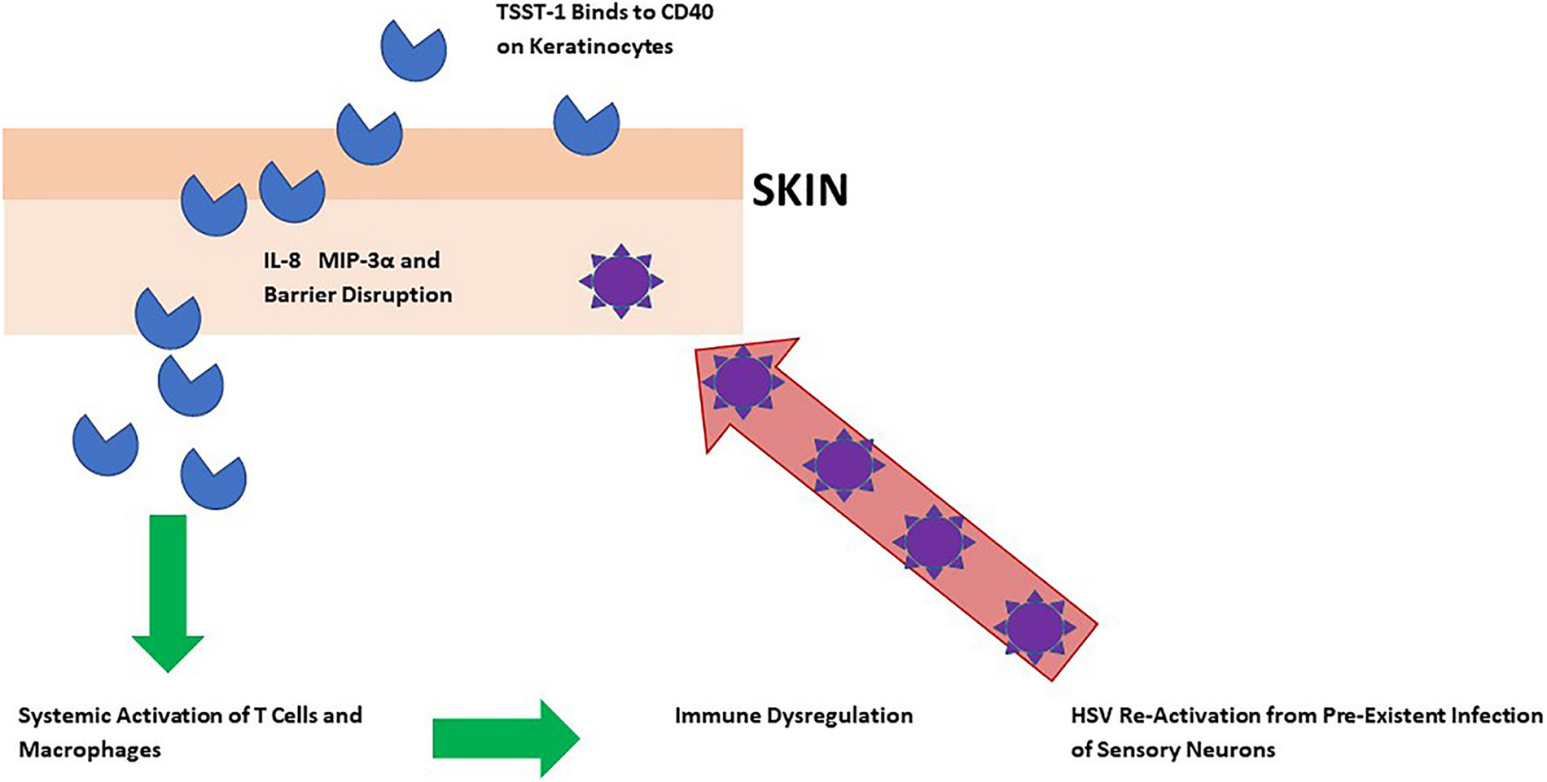
Model for the association of TSST-1 with ADEH.

Figure 4 provides a model for the association of TSST-1 (and to a lesser extent SEA, SEB, and SEC) with ADEH. TSST-1 is produced by S. aureus on the skin surface. The SAg binds to CD40 on keratinocytes with consequent upregulation of expression of chemokine genes. This leads to recruitment of immune system cells and harmful inflammation. Subsequently, TSST-1 systemically activates T lymphocytes and macrophages, resulting in general immune dysregulation. The resultant immune dysregulation facilitates reactivation of latent HSV infection in sensory neurons. HSV then also contributes to skin barrier disruption. The proposed reason for the lesser association of ADEH with SEA, SEB, and SEC is that these three SAgs may not bind CD40 as effectively as TSST-1. The reason other SAgs are not highly associated with ADEH is because they are produced in minute quantities.

## MATERIALS AND METHODS

### ADEH isolates.

Fifteen isolates of S. aureus were obtained in a blinded manner from the ADRN repository, maintained in the Schlievert laboratory, as coded by the ADRN. The isolates were from lesion skin. The patients were evaluated for EASI score. The isolates were evaluated for production of TSST-1, SEB, and SEC by PCR for gene presence (38) and production of the SAg proteins by quantitative antibody assays after growth in broth cultures of Todd-Hewitt (TH) medium (Difco Laboratories, Detroit, MI), at 37°C with 200-rpm shaking until stationary phase (39). Production of these three SAgs in ADEH was compared to production by recent isolates from typical AD not complicated by HSV infection (5) and from patients with cystic fibrosis, as two populations from non-ADEH conditions (6). When the code was broken to allow identification of the lesion ADEH isolates, two of the isolates were shown not to have the gene for or to produce TSST-1, SEB, or SEC. Since SEA is occasionally associated with nonmenstrual TSS (15), the two isolates were evaluated for SEA protein and for amount produced after growth in broth cultures, as described above for TSST-1, SEB, and SEC. The Schlievert laboratory maintains specific rabbit hyperimmune antisera raised against each of the four SAgs.

### Tissue culture cell lines.

Immortalized HVECs and human keratinocytes have been described in detail previously (13, 16, 33). The cell lines have cell surface markers and function similarly to primary cell lines. The isogenic HVEC line with CD40 knocked out through CRISPR/Cas9 technology has been described previously (13). All cell lines are maintained as −80°C stocks in the Klingelhutz and Schlievert laboratories. The cells are grown in keratinocyte serum-free medium (KSFM). HaCaT cells have been described previously (40, 41) and were maintained in Dulbecco’s modified Eagle’s medium (DMEM) supplemented with 10% fetal bovine serum. Vero cells were propagated in DMEM supplemented with 5% bovine calf serum.

### SAgs.

Highly purified TSST-1 was prepared from an RN4220 clone containing the TSST-1 gene by ethanol precipitation, resolubilization in pyrogen-free water, and thin-layer isoelectric focusing (42). The SAg thus purified was free of detectable contaminating pyrogens and other S. aureus virulence factors. TSST-1 was used in experiments at concentrations of 10 to 100 μg/ml. This amount is considered physiological since S. aureus strains may produce up to 15,000 μg/ml in thin films as might be represented by lesion AD skin.

Since SEA, SEB, and SEC are select agents of bioterrorism, RN4220 clones could not be used to prepare SEA, SEB, and SEC for use as controls in experiments and to prepare hyperimmune antisera. Instead, strain FRI722 was used for production of SEA, MNHO was used for production of SEB, and MNDON was used for production of SEC; all of these are naturally occurring strains without recombinant plasmids. SEA, SEB, and SEC were also purified by ethanol precipitation, resolubilization in pyrogen-free water, and preparative thin-layer isoelectric focusing (43).

### Herpes simplex virus preparations.

The properties of HSV-1(F) have been previously described (44). Viral stocks are maintained in the laboratory of author R. J. Roller. Virus was propagated, and infectious titers were determined by plaque assay on Vero cells.

### Multistep growth and spread of HSV-1(F).

HaCaT cells in confluent 24-well cultures were infected with 1,000 PFU of HSV-1(F) in a volume of 250 μl DMEM supplemented with 1% heat-inactivated calf serum (V medium) for 90 min at 37°C, at which point an additional 750 μl of V medium was added, and incubation was continued. At 12, 24, 36, 48, and 60 h after initiation of infection, 250 μl of culture medium was withdrawn for measurement of infectious virus and was replaced with an equivalent volume of fresh V medium. Infectious virus in samples was measured by plaque assay on Vero cells. Plaque size assays for determination of virus spread were performed as previously described (45).

### Immunological assays.

Quantification of the chemokine IL-8, as representative of chemokines produced by HVECs and keratinocytes, was performed with use of Quantikine kits from R&D Systems.

### Statistics.

Statistical procedures employed in these studies included Fisher’s exact test, means ± standard deviation (SD), and Student’s *t* test of unpaired, normally distributed data. In all studies, *P* < 0.05 was considered significant.
